# Une masse palpébrale révélant une fistule carotidocaverneuse

**Published:** 2012-11-13

**Authors:** Noureddine Oulali, Fayçal Moufid, Mohammed Khoulali, Rachid Sekhsoukh, Brahim Housni, Mohamed Rachid Ghailan

**Affiliations:** 1 Department of Neurosurgery, Medical School, University Mohammed First, Oujda, Morocco; 2 Department of Ophthalmology, Medical School, University Mohammed First, Oujda, Morocco; 3 Department of Anesthesia-healthcare, Medical School, University Mohammed First, Oujda, Morocco; 4 Department of Otolaryngology, Medical School, University Mohammed First, Oujda, Morocco

**Keywords:** Masse palpébrale, exophtalmie, fistule carotidocaverneuse, embolisation, eyelid mass, exophthalmia, carotid-cavernous fistula, embolization

## Abstract

Les auteurs rapportent une observation particulière, chez un homme de 48 ans ayant dans ces antécédents un traumatisme crânio-faciale grave il y'a dix ans, qui présente depuis huit mois une masse palpébrale droite, avec une discrète exophtalmie et hémorragie sous conjonctivale, l'angio IRM a permis de confirmer le diagnostique d'une fistule carotidocaverneuse à haut débit, qui est responsable de cette symptomatologie. L'objectif de cet article est de mettre la lumière sur cette pathologie rare, en insistant sur les particularités cliniques de présentation d'une part, et sur le plan thérapeutique d'autre part, en exposant l'intérêt de la radiologie interventionnelle.

## Introduction

La fistule carotidocaverneuse est une communication anormale entre la carotide interne et le sinus caverneux, c'est la forme la plus fréquente des fistules artério veineuses cérébrales post-traumatiques. Il s'agit d'une pathologie rare. Dont l'étiologie traumatique est retrouvée dans 75% des cas. Elles sont dues à une communication directe entre la portion intra-caverneuse de la carotide interne et le sinus caverneux (brèche traumatique de l'artère) responsable d'une fistule à haut débit [1]. La symptomatologie clinique associe classiquement une exophtalmie pulsatile, un souffle perçu par le patient ainsi qu'une dilatation des veines épisclérales avec chémosis [2]. Cette pathologie, peu fréquente, est une urgence thérapeutique car elle met en jeu le pronostic vital et visuel. L'attitude thérapeutique principale actuelle est axée sur la neuroradiologie interventionnelle.

## Patient et observation

**Figure 1:**
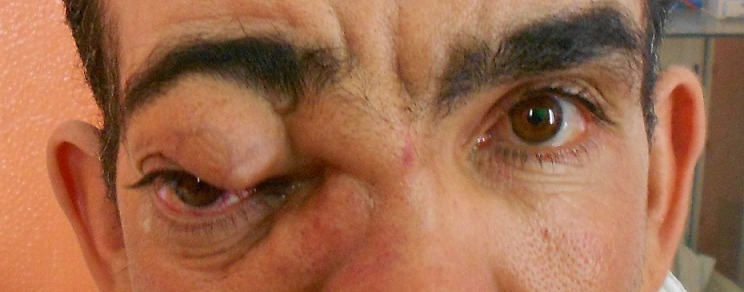
Vue de face montrant une masse palpébrale droite "en tête de méduse"

**Figure 2:**
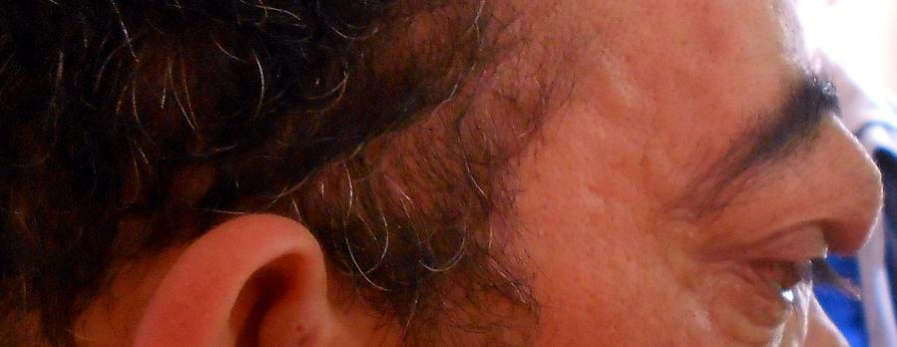
Vue de profil de la masse palpébrale

**Figure 3:**
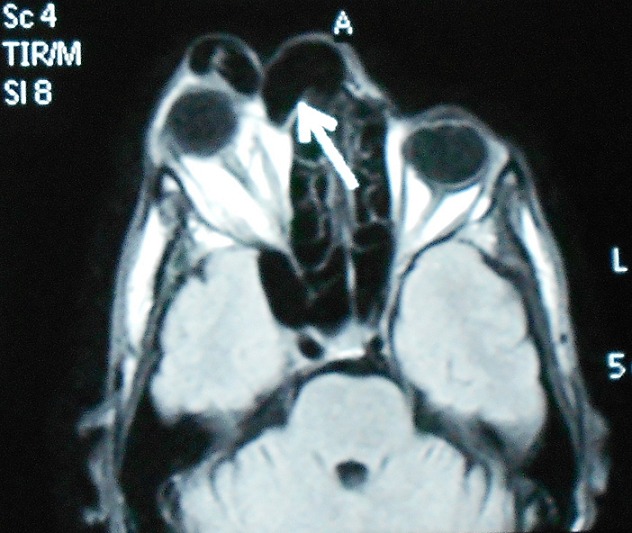
IRM orbito-encéphalique séquence T1 montrant une exophtalmie droite

**Figure 4:**
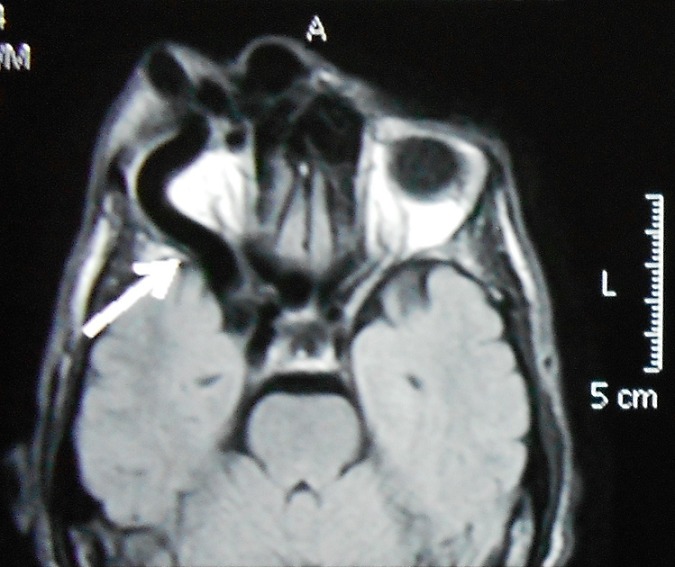
Angio-IRM séquence veineuse montrant un élargissement de sinus caverneux à droite

**Figure 5:**
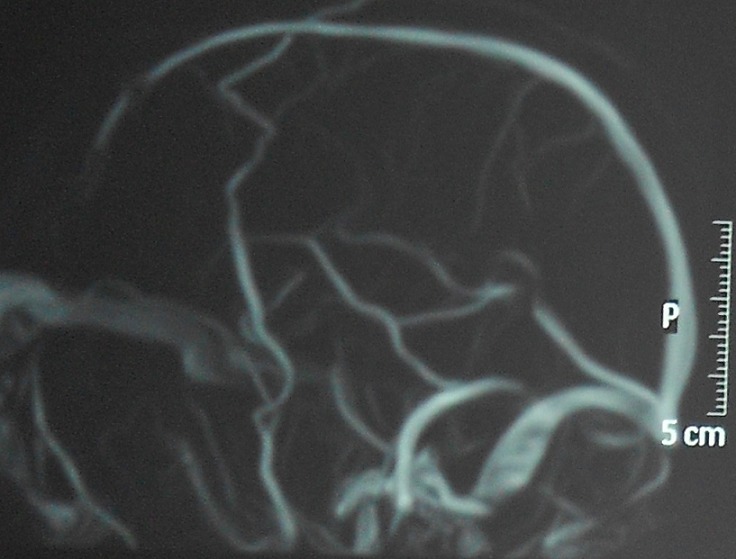
Angio-IRM séquence artérielle montrant une artérialisation du sinus caverneux

**Figure 6:**
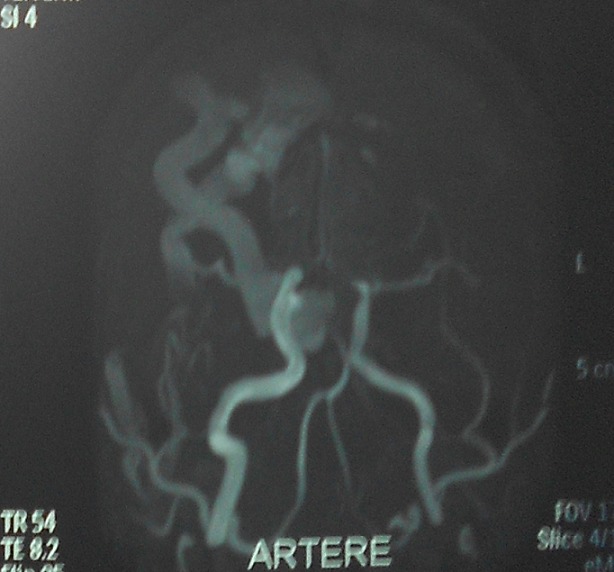
IRM séquence T1 montrant une dilatation de la veine ophtalmique supérieure (flèche)

Un homme de 48 ans ayant comme antécédent un traumatisme crânio-faciale grave Il y'a dix ans, qui présente depuis 08 mois, une masse palpébrale droite qui augmente progressivement de volume (Figure 1), une discrète exophtalmie et une hémorragie sous conjonctivale (Figure 2). L'examen clinique trouve une masse battante, un chémosis. L'auscultation orbitaire et temporale trouve un souffle systolodiastolique, qui disparait à la compression de l'artère carotide homolatérale au niveau cervicale, par contre l'examen neurologique et strictement normal. Un bilan neuroradiologique fait d'une angio-IRM encéphalique et orbitaire (Figure 3, 4) a confirmé le diagnostique d'une géante fistule carotidocaverneuse à haut débit (Figure 5, Figure 6). Le patient a été adressé à l'unité de neuroradiologie interventionnelle pour une prise en charge endovasculaire par embolisation.

## Discussion

La fistule carotidocaverneuse directe, est une communication anormale entre l'artère carotide interne et le sinus caverneux. Elle correspond généralement à une fistule à haut débit même si, en cas d'adaptation veineuse secondaire, un bas débit est possible [2]. Il s'agit d'une pathologie rare dont l'étiologie traumatique est retrouvée dans 75% des cas. La cause en est le plus souvent un traumatisme crânien chez un homme jeune [3]. Les mécanismes incriminés dans la genèse des FCC post-traumatiques sont de trois ordres: un arrachement sous l'effet du choc de rameaux artériels intracaverneux du siphon carotidien; une rupture d'un anévrysme préexistant ou bien une plaie artérielle secondaire au déplacement d'un fragment osseux dans les grands fracas craniofaciaux, et notamment les fractures de la base du crâne [4, 5].

Plus rarement, il peut s'agir d'une fistule spontanée par la rupture d'un anévrisme intracaverneux sur un terrain prédisposant comme la maladie d'Ehlers-Danlos. Quelques cas de dysplasie fibromusculaires ont également été rapportés [6].

Le délai d'apparition des symptômes est variable de quelques semaines à quelques mois, voire plusieurs années comme c'est le cas de notre observation, le tableau clinique est aigu où rapidement progressif. Il associe classiquement une exophtalmie pulsatile avec un souffle synchrone au pouls, L'auscultation de la région périorbitaire et temporale retrouve un souffle intracrânien systolodiastolique disparaissant à la compression manuelle de l'artère carotide homolatérale au niveau du cou [5], une artérialisation des vaisseaux conjonctivaux qui prennent un aspect en « tire-bouchon », un chémosis et une dilatation du réseau veineux de la paupière supérieure (aspect de méduse) et une paralysie oculomotrice. Les acouphènes observés chez ces patients correspondent à la perception de la turbulence du flux vasculaire dans la fistule. Les manifestations cliniques sont le plus souvent unilatérales et homolatérales à la fistule, mais peuvent parfois être bilatérales voire controlatérales puisque les deux sinus caverneux communiquent entre eux.

Une fois le diagnostic de fistule évoqué, la tomodensitométrie orbitaire ou l'IRM permettent de rechercher de signes indirects comme l'élargissement du sinus caverneux, la dilatation de la veine ophtalmique supérieure.

La visualisation de la fistule carotidocaverneuse est possible sur une angio-IRM dynamique ou par une artériographie qui reste l'examen de référence pour le diagnostic et qui permet, de planifier la prise en charge thérapeutique [5, 7].

L'IRM encéphalique, coupe passant par le plan neuro-oculaire [7], sans injection de produit de contraste, confirme l'exophtalmie et la dilatation de la veine ophtalmique supérieure qui est le signe indirect le plus évocateur de fistule carotidocaverneuse. L'IRM orbitaire en séquence T1 objective une dilatation de la veine ophtalmique supérieure (hypersignal si thrombose, hyposignal si flux rapide), un élargissement du sinus caverneux homolatéral. En séquence T2, elle permet une meilleure visualisation du sinus caverneux [5, 7].

Barrow et al. [8] ont proposé en 1985 une classification des fistules carotido-caverneuses en 4 types: **type A**: fistules carotido-caverneuses à plein canal, à haut débit; **type B**: fistules durales à bas débit, alimentées par les branches de la carotide interne intra-caverneuse; **type C**: fistules durales à bas débit, alimentées par les branches à destinée caverneuses de la carotide externe; **type D**: fistules durales à bas débit, alimentées à la fois par les branches de la carotide interne et de la carotide externe. Cette pathologie, peu fréquente, est une urgence thérapeutique car elle met en jeu le pronostic vital et visuel [9], et nécessite une étroite collaboration entre radiologues, neurochirurgiens et ophtalmologistes.

Le traitement actuel des FCC se fonde sur la neuroradiologie interventionnelle [5]. Son principe est l'occlusion de la fistule par largages des ballonnets intravasculaires détachables ou des spires métalliques (coils) tout en respectant la perméabilité de l'axe carotidien. L'abord peut se faire par voie artérielle (fistule basse et postérieure) ou voie veineuse rétrograde (fistule haute et antérieure) [1, 4]. Le traitement neurochirurgical par ligature artérielle garde sa place en cas d'échec de la thérapeutique endovasculaire.

## Conclusion

La fistule carotido-caverneuse est une pathologie rare, mais non exceptionnelle, plus fréquemment rencontrées lorsqu'il existe une fracture de la base du crâne. Le diagnostic clinique est évoqué sur des signes ophtalmiques et orbitaires. La visualisation des signes directs ou indirects sur l'angio-TDM et l'angio-IRM cérébrales doit conduire à réaliser une angiographie cérébrale, examen de référence, à la fois diagnostique et thérapeutique. La neuro-endovasculaire interventionnelle est indiscutablement le traitement de premier choix. Les indications de sacrifice chirurgical de la carotide se résument actuellement aux échecs des techniques neuro-endovasculaire.
